# More than a reposition tool: additional wire cerclage leads to increased load to failure in plate osteosynthesis for supracondylar femoral shaft fractures

**DOI:** 10.1007/s00402-020-03586-1

**Published:** 2020-08-27

**Authors:** Christopher Bliemel, Dan Anrich, Tom Knauf, Ludwig Oberkircher, Daphne Eschbach, Antonio Klasan, Florian Debus, Steffen Ruchholtz, Martin Bäumlein

**Affiliations:** Center for Orthopaedics and Trauma Surgery, University Hospital Giessen-Marburg, Baldingerstrasse, 35043 Marburg, Germany

**Keywords:** Supracondylar femoral fracture, Polyaxial angular stable plate osteosynthesis, Wire cerclage, Biomechanical analysis, Load to failure

## Abstract

**Introduction:**

Surgical treatment of supracondylar femoral fractures can be challenging. An additional wire cerclage is a suggested way to facilitate fracture reduction prior to plate osteosynthesis. Denudation to the periosteum remains a problematic disadvantage of this procedure.

This study analyzed the effect of an additional wire cerclage on the load to failure in plate osteosynthesis of oblique supracondylar femoral shaft fractures.

**Materials and methods:**

On eight pairs of non-osteoporotic human femora (mean age 74 years; range 57–95 years), an unstable AO/OTA 32-A2.3 fracture was established. All specimens were treated with a polyaxially locking plate. One femur of each pair was randomly selected to receive an additional fracture fixation with a wire cerclage. A servohydraulic testing machine was used to perform an incremental cyclic axial load with a load to the failure mode.

**Results:**

Specimens stabilized with solely plate osteosynthesis failed at a mean load of 2450 N (95% CI: 1996–2904 N). In the group with an additional wire cerclage, load to failure was at a mean of 3100 N (95% CI: 2662–3538 N) (*p* = 0.018).

Compression deformation with shearing of the condyle region through cutting of screws out of the condylar bone was the most common reason for failure in both groups of specimens. Whereas axial stiffness was comparable between both groups (*p* = 0.208), plastic deformation of the osteosynthesis constructs differed significantly (*p* = 0.035).

**Conclusions:**

An additional wire cerclage significantly increased the load to failure. Therefore, an additional cerclage represents more than just a repositioning aid. With appropriate fracture morphology, a cerclage can significantly improve the strength of the osteosynthesis.

## Introduction

Supracondylar femoral fractures remain challenging to treat due to complex anatomical conditions and a vulnerable patient collective, either polytraumatized or geriatric [1, 2]. For the treatment of such extra-articular distal femoral fractures, apart from retrograde femoral nails, polyaxial angular stabile plates are the most common type of fixation devices [3].

While reconstructions of length, axis, and rotation are of major importance in comminuted distal femoral fractures, in two-part fractures, a good fracture repositioning with adequate support of the fragments is advantageous to obtain a proper fracture healing and satisfying clinical results [4].

**Fig. 1:**
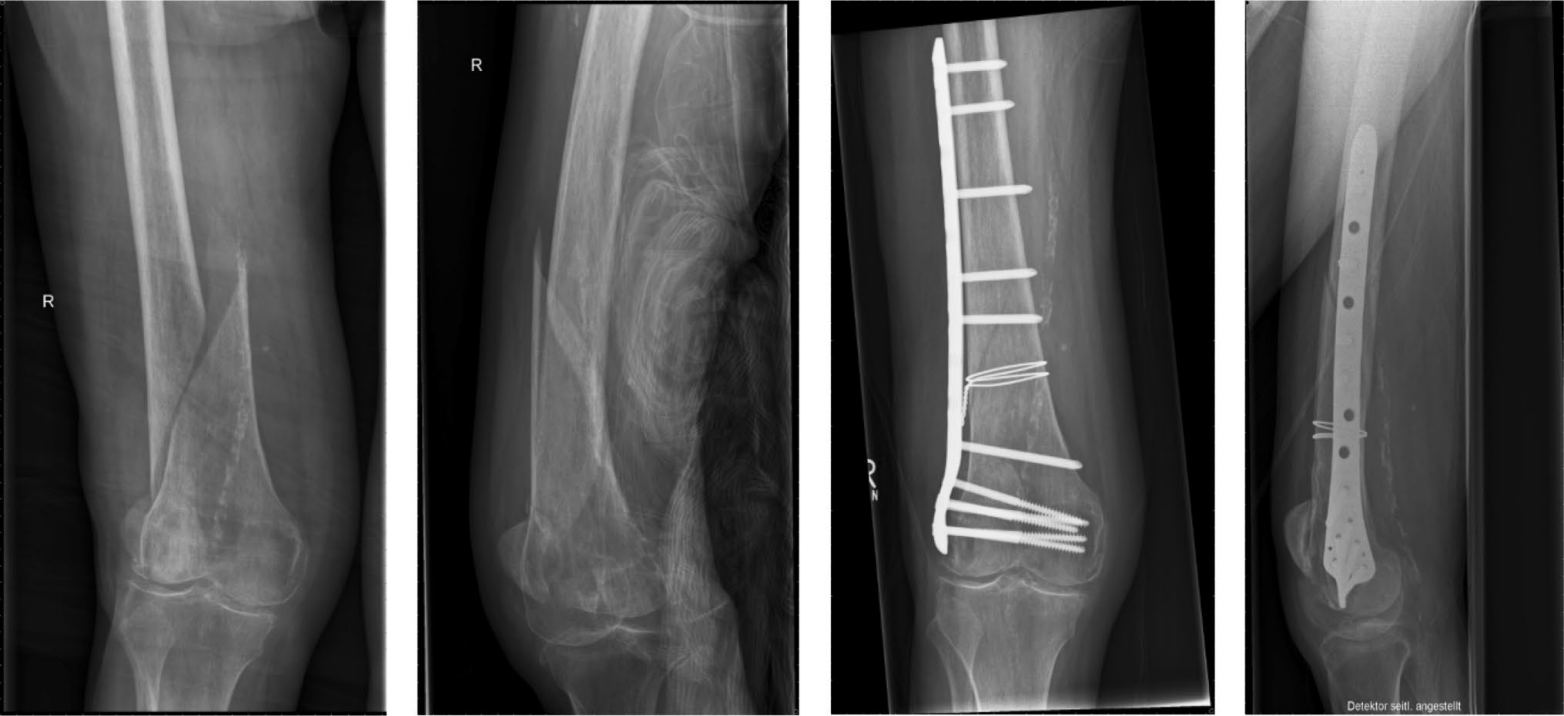
Clinical case of a 78-year-old woman with a 32-A2.3 distal femoral fracture. The fracture was surgically treated with a direct fracture repositioning using a double-looped wire cerclage and a retrograde inserted, polyaxially angular plate osteosynthesis

In this context, in addition to a polyaxially angular stabile plate, the prior use of a wire cerclage for an initial anatomical reduction of extra-articular two-part spiral or oblique distal femoral fractures has been shown to be beneficial [5, 6] (Fig. 1). Despite this above-named positive effect of a wire cerclage as a tool for an anatomical fracture reduction, denudation of the periosteum and disturbance of the blood supply in the fracture region remain problematic drawbacks of this procedure posing an increased risk for nonunion [7].

To investigate whether a wire cerclage remains just a simple tool for an anatomical fracture repositioning prior to plate osteosynthesis or also contributes to the overall stability of the osteosynthesis construct, a biomechanical study was conducted.

It was presumed that the wire cerclage would provide a better compression of the bone fragments supporting the stability in the fracture region. Therefore, it was hypothesized that the load to failure of osteosynthesis constructs supplied with an additional wire cerclage would be increased compared to that of a simple fracture fixation with a polyaxially angular plate osteosynthesis.

## Materials and methods

### Specimens

Eight pairs of matched, adult human femora were available for this biomechanical study. The femora were provided from the Institutes of Anatomy and Cell Biology of Philipps University, Marburg, Germany. All the donors gave their written consent by their own free will for the use of their body for research purposes. A positive ethics approval was obtained by the local ethics committee (AZ 127/18).

### Assessment of bone quality

Prior to testing, the conventional anterior–posterior and medial–lateral radiographs were taken of each femur to search for pre-existing pathologies or fractures. Bone mineral density of the hip was measured in each femur using Dual-energy X-ray Absorptiometry (DXA).

### Implants

The Non-Contact Bridging plate for Distal Femur (NCB^®^-DF, Zimmer GmbH, Winterthur, Switzerland) was used exclusively for this study. The NCB^®^-DF is a polyaxially locking device that is anatomically performed for right and left femurs to ensure an optimum fit to the lateral cortex. In the present study, solely NCB^®^-DF nine-hole plates, which are made of titanium alloy, were used. All osteosyntheses were performed by the same study surgeon (BM).

Due to technical reasons in a laboratory setting with missing soft tissue, the plate osteosynthesis was fixed to the bone prior to performing an osteotomy gap. Thereafter, a wire cerclage was attached in all specimens assigned to group 1.

The NCB-DF plate was attached laterally to the femur, 1.5 cm proximal to the lateral articular surface. For this purpose, the fixation of the plate was accomplished with a blunt bone reduction clamp. Shaft fixation of the plate was achieved with four bicortical, fully threaded 5 mm cortical screws, and drilled with a 4.4 mm tip. For condyle fixation, five monocortical, partially threaded 5 mm cancellous screws were used with a drill hole of 2.5 mm.

### Fracture model

After fixation of the NCB-DF plates to the femur, a standardized femur osteotomy was created using an oscillating saw. As a fracture model, a supracondylar oblique femoral fracture was simulated (32-A2.3 in the AO/OTA classification). Therefore, after measurement of the individual condylar width of each femur, an oblique osteotomy was performed beginning at the level of one condylar length proximal to the joint line. The fracture gap extended from the distal anterior corticalis, at an angle of 70° in relation to the diameter of the femur, to the proximal posterior corticalis of the femur. This kind of osteotomy was chosen to prevent that the shearing fracture fragments could support themselves on the laterally attached plate, under the applied load during the testing.

After the creation of the osteotomy, specimens of each pair of samples were randomly assigned by coin toss into two different groups.

**Fig. 2:**
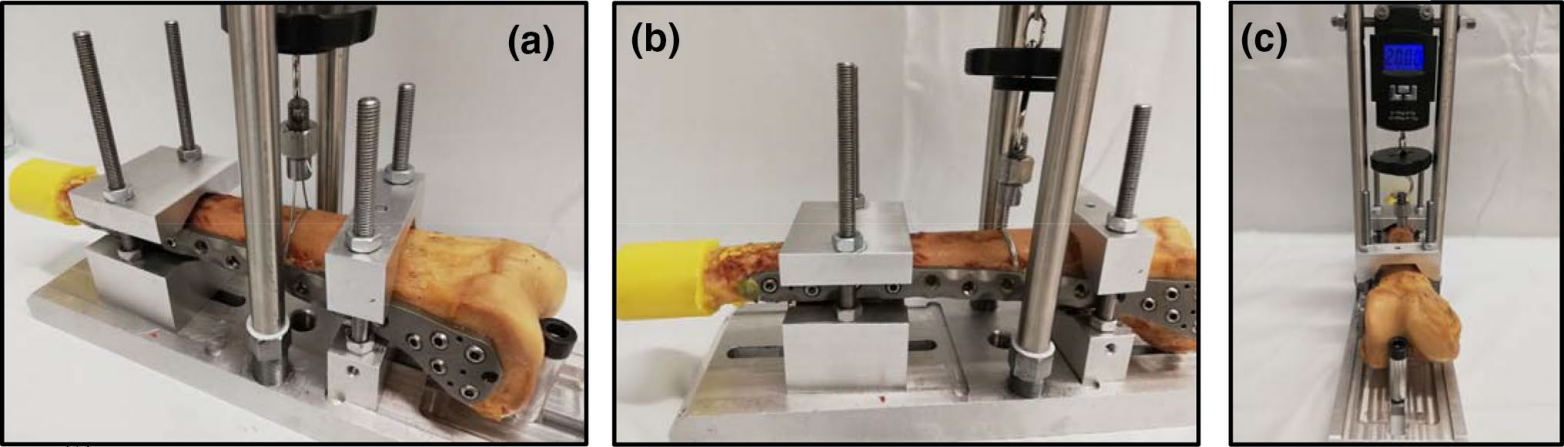
All femora assigned to group 1 were fixed in a custom-made torque wrench (**a**). A wire cerclage was applied in the center of the oblique fracture and tightened under laboratory conditions (**b**), thereby ensuring that the cerclage was attached with a force of 200 N (**c**)

**Fig. 3:**
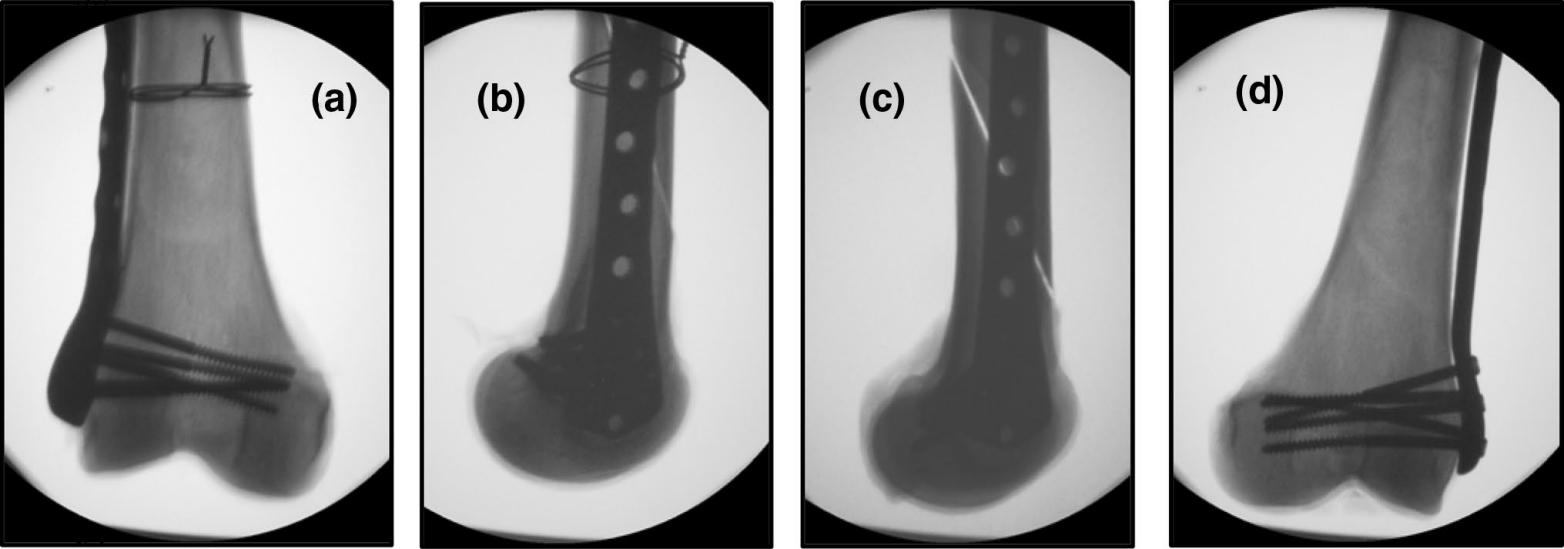
Typical postoperative X-ray pictures showing a pair of samples with an NCB-DF plate osteosynthesis and an additionally attached double-looped wire cerclage in the right femur (**a** and **b**) (the same femur is shown in Fig. 2). The left femur only received fixation with an NCB-DF plate (**c** and **d**)

All femora assigned to group 1 received further stabilization with an additional double-looped wire cerclage that was centered in the area of the oblique fracture. The cerclage consisted of steel wire with a thickness of 1.25 mm. The cerclage was tightened with the help of a torque wrench, thereby ensuring that the cerclage was attached with a force of 200 N (Fig. 2a–c). All femora assigned to group 2 remained without a further wire cerclage. Figure 3a–d illustrates the fracture fixation in the two groups, either with an additional supply of a wire cerclage (group 1) or not (group 2).

### Biomechanical testing

**Fig. 4:**
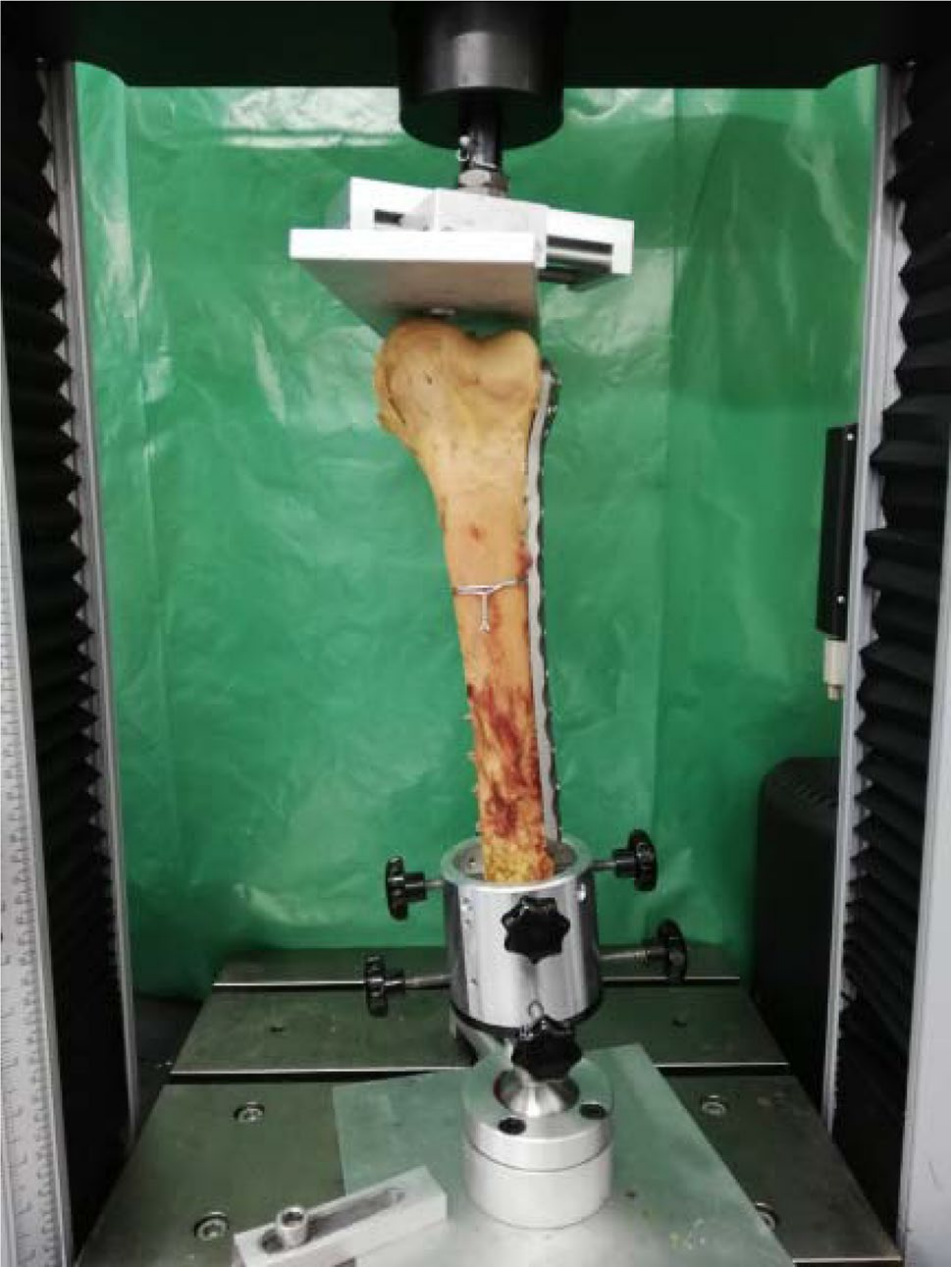
Test setup demonstrating force application with a metal plate movable in two directions. The femoral shaft was statically fixed in an anatomical 5–7° valgus position. Force was applied in a retrograde manner

Each femur was truncated 6 cm proximal to the plate osteosynthesis. Afterwards, the proximal part of each specimen was embedded into a special form cup using Technovit 3040 (Heraeus, Wehrheim, Germany). Using a custom-made holding device, the femora were positioned upside down in an Instron 5566 universal servohydraulic testing machine (Instron Cor., Darmstadt, Germany). As a pressure device, a metal plate, movable in two directions (anterior–posterior and medial–lateral), was used. The femur condyles were positioned parallel to the horizontal axis. By this means, the weight-bearing axis along the femur shaft axis was accomplished (Fig. 4).

To compress the specimen and to avoid deformation artifacts in the bone construct on each femur, a preload of 100 N was applied. According to a standardized protocol, subsequently, each femur was subjected to cyclic loading. The test sequence started with 500 cycles at 800 N. The load was continuously increased at increments of 200 N every 500 cycles until a failure of osteosynthesis was achieved. To measure construct failure, sudden loss of measured force (> 30%) and major deformation of the bone construct (> 20 mm) were defined. The testing was conducted in a displacement control mode.

### Data collection and statistical analysis

Loading (N), deformation (mm), and the number of cycles were recorded at 100 ms intervals using instrument-specific Bluehill Software.

Plastic deformation, as a measure of irreversible deformation under the influence of force, was calculated by subtracting the initial construct height from displacement present after reaching the endpoint once the load was removed. The stiffness of the specimens was calculated from the compression set under the applied loading forces.

Based on load to failure values reported in the previous biomechanical studies on distal femoral fractures, an a-prior power analysis was performed [4, 8]. Based on a power of 0.80 and an alpha error of 0.05, a sample size of eight pairs of femora was calculated. A clinically relevant difference was defined if the construct failure occurred in 85% of osteosynthesis with solely angular stable plate osteosynthesis and up to 30% in the specimens with the combined plate and wire cerclage osteosynthesis at a load of 2800 N.

Statistical analysis was performed using IBM SPSS statistics 24 (Statistical Package for the Social Science, IBM Cooperation, Armonk, New York, USA). Due to low sample number, the Wilcoxon rank-sum test (non-parametric test) for paired samples was used for data analysis. Statistical significance was set at *p* < 0.05.

## Results

The samples originated from one female and seven male adults, with an average age of 74 years (range 57–95 years). The exact characteristics of each specimen are shown in Table 1.

**Table 1:** Characteristics of the specimens used for biomechanical testing

Pair of specimens	Femur with cerclage	Gender	Age (years)	Height (cm)	Weight (kg)	Diameter of condyles		T score	
						Left femur (cm)	Right femur (cm	Left femur	Right femur
1	Right	Male	71	180	95	8.7	9.2	− 0.7	0.6
2	Left	Male	79	168	82	8.6	9.0	− 1.2	− 1.6
3	Left	Male	75	168	75	9.1	9.3	− 0.5	− 0.9
4	Left	Female	57	158	70	8.3	8.1	− 1.4	− 1.3
5	Left	Male	73	162	71	8.8	9.2	− 2.2	− 2.1
6	Left	Male	69	174	90	9.2	8.9	− 1.2	− 1
7	Right	Male	72	183	100	9.3	9.4	− 0.5	− 0.3
8	Right	Male	95	175	90	9.3	9.3	− 1.7	− 1.2

### Bone quality assessment

DXA showed intact bone quality in all the tested specimens, with a mean T score of − 1.08 (CI: − 0.37 to − 1.78). Bone mineral density was comparable in all the matched pairs of cadaver femora (*p* = 0.106). Preoperative X-rays revealed no pre-existing pathologies or fractures in any of the examined specimens.

### Cyclic testing and failure mode

**Fig. 5:**
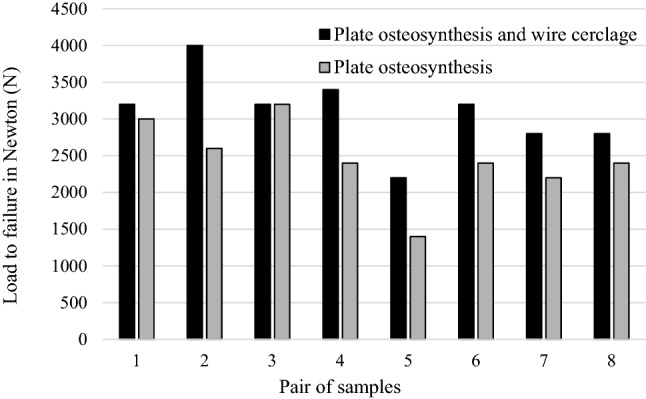
Failure loads based on the cyclic loading tests, comparing plate osteosynthesis with and without additional wire cerclage

The entire examined specimens withstood at least a load of 1200 N. The mean compressive forces leading to failure were 2450 N (95% CI: 1996–2904 N) in the group with solely angular stable plate osteosynthesis and 3100 N (95% CI: 2662–3538 N) in the group with a combination of angular stable plate osteosynthesis supported by an additional wire cerclage. The difference in the values for the load to failure was statistically significant (*p* = 0.018) (Fig. 5).

**Fig. 6:**
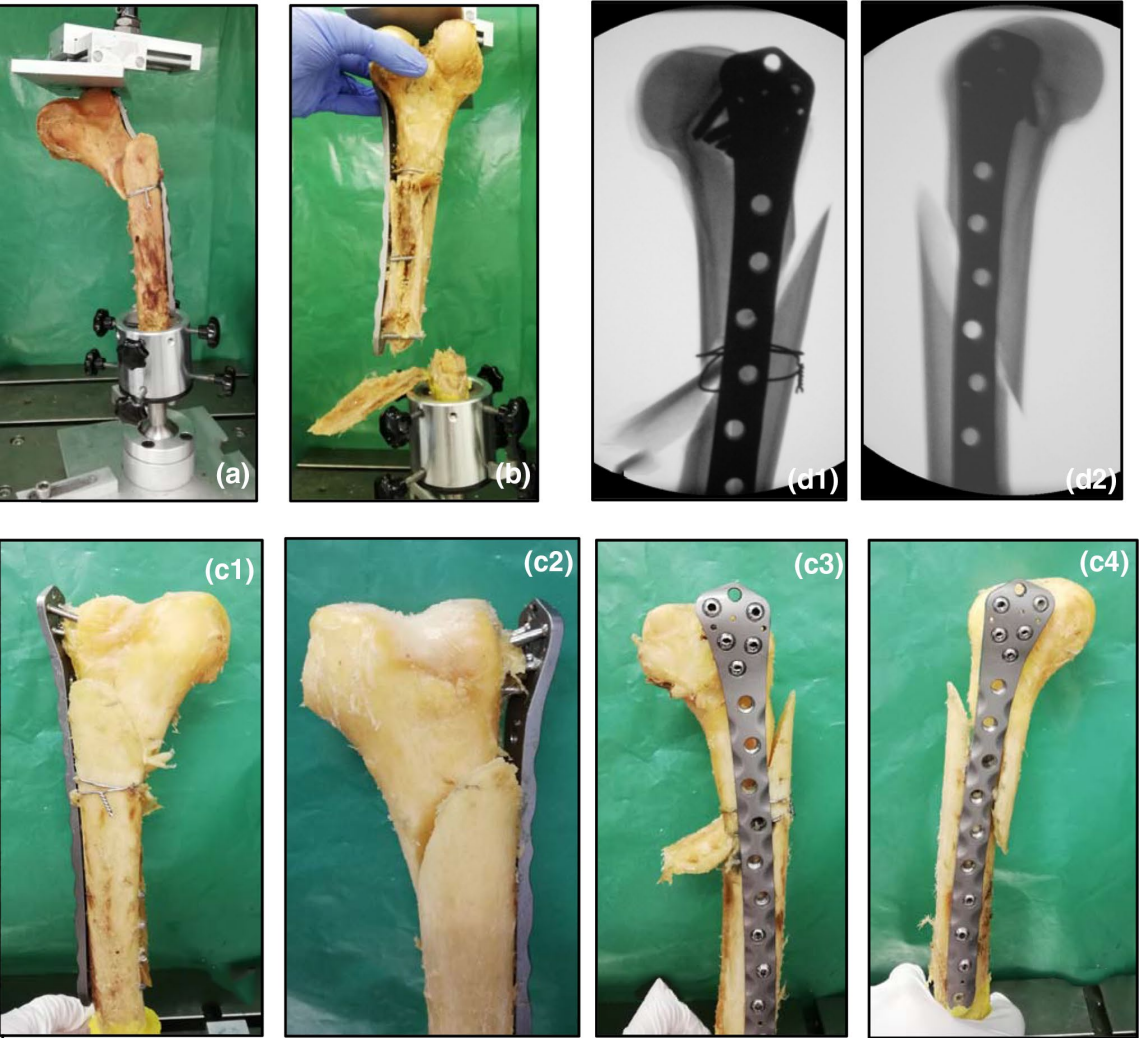
Photographs showing the different types of construct failure. Reasons for failure included irreversible deformation of the osteosynthesis plate (**a**) and multifragmentary fracture of the femoral shaft (**b**). Most common in both groups was failure due to cutting out of the screws (**c** 1–4) with shearing away of the condylar region. In most specimens with wire cerclage, the dorsal buttress broke under the cerclage at this failure mode. (**d** 1 and 2) illustrate the corresponding X-ray pictures to the specimen shown in (**c** 2 and 3)

In both groups, the same three different failure modes occurred. Deformation of the construct due to irreversible bowing of the plate osteosynthesis occurred twice in the conventional group and once in the group with an additional wired cerclage (Fig. 6a). Multifragmentary femoral shaft fracture with cutting out of the shaft screws occurred once in both groups (Fig. 6b). Shearing of the condylar region with cutting out of the condylar screws was the most frequent reason for failure in both groups, with six incidences in the cerclage group and five in the conventional group (Fig. 6c). In all specimens with wire cerclage, the dorsal buttress broke under the cerclage, while just one out of eight specimens in the conventional group experienced this type of breakage. Figure 6d shows the corresponding X-ray pictures to the failure mode that most frequently occurred.

### Axial stiffness and plastic deformation at a load of 1200 N

All specimens withstood at least a cyclic force of 1200 N. Therefore, a further analysis of stiffness and plastic deformation was performed at this loading step for all examined specimens.

**Fig.7:**
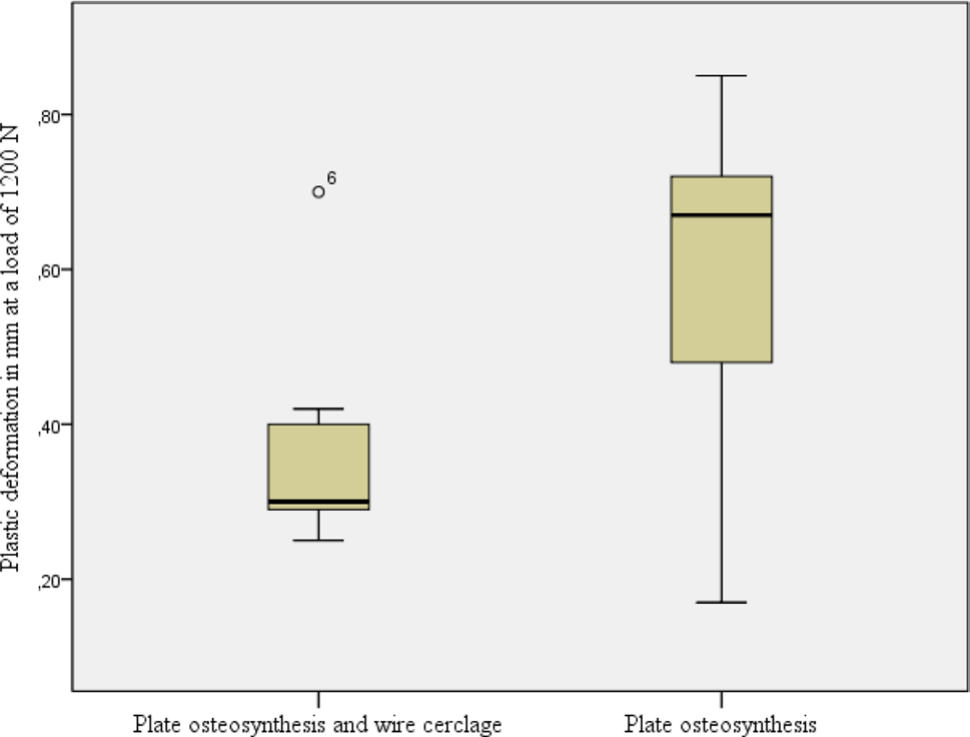
Plastic deformation of the two different osteosynthesis constructs at a load of 1200 N

Stiffness of the osteosyntheses constructs was comparable at this load between the group with osteosyntheses with an additional wire cerclage (mean 1.49 kN/mm; 95% CI: 1.04–1.94 kN/mm) and the group with only a plate osteosynthesis (mean 2.00 kN/mm; 95% CI: 1.11–2.90 kN/mm) (*p* = 0.208). Analysis of the irreversible, plastic deformation of the osteosynthesis construct revealed significant differences between the two groups (*p* = 0.035). The data analysis illustrated in Fig. 7 shows a lower range of variability in terms of plastic deformation if the osteosynthesis was enhanced with an additional wire cerclage (mean 0.37 mm; 95% CI 0.22–0.51 mm) compared to the group with only a plate osteosynthesis (mean 0.60 mm; 95% CI 0.38–0.81 mm).

## Discussion

This biomechanical study analyzed the stability-enhancing effect of a wire cerclage in angular stable plate osteosynthesis for supracondylar femoral shaft fractures. The major study results indicate that an additional wire cerclage applied for primary fracture reposition not only supports an anatomically fracture reduction but also contributes significantly to an increase in load to failure. This increase in stability was accompanied by minor plastic deformation in the cerclage group and similar stiffness between the two osteosynthesis groups.

Underlying the multimorbidities of most geriatric trauma patients, partial weight-bearing is extremely difficult for them to implement, if not even impossible to practice [9]. Therefore, such patients depend on immediate full-weight-bearing. In this context, several studies on geriatric trauma patients have shown that postoperative early full-weight-bearing mobilization significantly reduces perioperative complications and mortality [10, 11]. Modern polyaxially angular stable plates offer a crucial advantage in terms of stability and, therefore, in the treatment of geriatric trauma patients with distal femoral fractures [12–14].

Despite the reduction in complications with the use of angular stable implants, the loss of fracture reduction under full-weight-bearing due to tension forces of the muscles remains an important issue in the treatment of geriatric supracondylar femoral fractures [15].

In this context,especially in two-part oblique and spiral fractures, the use of an additional wire cerclage prior to plate osteosynthesis represents a proven tool for fracture repositioning, posing the possibility for an even higher stability of the entire osteosynthesis. The implant industries employ specially developed cable tensioners for this procedure. In this regard, prior examinations of different options for the creation of a wire cerclage have been conducted by different working groups [16–18]. The present biomechanical investigation incorporated the most crucial aspects of those previous examinations. According to the recommendations of Lenz et al., a double-looped wire cerclage was used tightened at a tension of 200 N following the suggestions of Harnroongroj [17, 18]. For an optimum stability of the 1.25 mm cerclage, the twisted cerclage lock was bent forwards, and the protrusions were then cut, as recommended by *Wähnert and co-workers* [16]. Following this application recommendation, the additional wire cerclage significantly increased the load to failure and, therefore, contributed to the overall stability of the osteosynthesis construct. The findings of the present study are, therefore, in line with the previous related examinations reporting on increased load to failure in nail osteosynthesis for subtrochanteric femoral fractures and in primary hip arthroplasty [19, 20].

This increase in stability, as shown in the present examination, was accompanied by a significant reduction in the plastic deformation of the construct itself. The cerclage, positioned in the middle of the fracture, significantly contributed to the stabilization of the osteotomy gap by absorbing compression forces and, therefore, led to a noticeable reduction of plastic deformation of the entire osteosynthesis construct. The results of the present investigation are consistent with those of other investigations on wire cerclages conducted by Müller et al. They proved that an additional wire cerclage could reduce plastic deformation in intramedullary nail osteosynthesis conducted for subtrochanteric femoral fractures, though without statistical significance due to too low sample size [20].

Despite these load-increasing effects, the application of cerclages for fracture fixation is considered critical in the literature due to a strangulation of periosteal vascularization, which could result in bone necrosis and non-unions [21]. In the present study in principle, a minimally invasive procedure (group 2) was compared with a procedure that can significantly impair the periosteal blood flow (group 1). Especially because it is well known that a minimally invasive inserted plate osteosynthesis significantly minimizes the risk of non-unions, the procedure performed by Lee et al. using additional percutaneous cerclage wiring in the treatment of distal femur fractures treated with the additional plate osteosynthesis could be an interesting option [22]. Despite this certain treatment option, three different mechanisms are accused to be responsible for an uneventful bone healing. Apart from the direct compressive contact of the cerclage to the bone, the compression of periosteal blood vessels and the sliding of the cerclage on the bone surface, the so-called ‘Gigli saw effect’, have been suggested to be responsible [6].

To prevent such negative outcomes, a careful surgical technique seems to be crucial for the preservation of the periosteal blood supply [23]. Thus, the focus should lie on the avoidance of periosteal devitalization through an extended stripping of soft tissue while exposing the fracture prior to reduction. Therefore, some authors reporting on impaired fracture healing following cerclage application suggest that it is less likely the compression of vital bone by the cerclage than the devascularization of the bone during fracture exposition leading to nonunion [21, 24]. This assumption is in line with several publications in the current literature reporting on clinical results using wire cerclages. Angelini and Battiato reported on a series of 54 cases treated with different techniques associated with a low-contact wire cerclage for femoral fracture. The cerclages used in their study had spheres designed to minimize the contact area at the cerclage to bone interface. At a mean follow-up of 10.5 months, fracture healing was achieved in 71% of their patients, with four patients having developed a nonunion [6]. Lee et al., examined data retrospectively collected over a 7-year period. In their monocenter cohort study, they included a total of 101 patients with oblique, spiral, or spiral wedge-type distal femoral fractures. Exclusive locking plate fixation was performed in 46 patients, while locking plate fixation with an additional cerclage wiring was performed in 55 patients. Clinical and radiological outcomes between the two groups were shown to be equal without increased complications, such as time to fracture union [25]. Kennedy and co-workers reported on 17 patients with subtrochanteric femoral fractures treated with a long cephalomedullary nail and an additional cerclage. They described the area of contact between a cerclage and the bone to be rather narrow; therefore, its effect on the osseous blood supply was negligible. Nevertheless, with respect to bone biology, the use of one or at most two cerclages is a viable method to ensure the biomechanical advantages of a secure anatomical reduction afforded by circumferential cerclages and the disadvantages of excessive periosteal stripping of the fracture region.

### Limitations

Despite a careful design, the present study still has some limitations. In this context, the in vitro setup, in which only axial loading of the specimen was tested, must be recognized. These restrictions of the loading conditions, in which torsional and bending loads were not considered, could have influenced the results with regards not only to failure mode but also to failure load. In addition, due to the in vitro design, conclusions can only be drawn concerning load-bearing capacity, but not in terms of the occurrence of an expected fracture healing. Finally, the use of formaldehyde embalmed human cadaver bones has to be mentioned, posing a risk in terms of comparability of the present biomechanical results to actual failure modes in real-life patients. In this context, there are controversies in the current literature concerning the authenticity of loading tests performed with formaldehyde preserved cadaveric bones. While some authors point out organic changes through the preservation process in cadaveric bones [26], Topp et al. could not find any significant differences between fresh frozen and embalmed femora concerning pull-out forces of cancellous and cortex screws and axial load until failure [27].

## Interpretation

This biomechanical stress analysis detected an increased load to failure in two-part distal femoral shaft fractures when a wire cerclage was combined with an angular stable plate osteosynthesis. Therefore, the application of an additional wire cerclage in two-part distal femoral shaft fractures is recommended, particularly in elderly patients for whom postoperative full-weight-bearing is of crucial importance.
